# Hub genes identification for diagnosing Alzheimer's disease in patients with Crohn's disease

**DOI:** 10.1016/j.gendis.2024.101476

**Published:** 2024-11-30

**Authors:** Pei Liu, Xiaoying Yao, Hongyan Wang

**Affiliations:** a Shanghai Key Laboratory of Metabolic Remodeling and Health, Institute of Metabolism and Integrative Biology, Fudan University, Shanghai 200438, China; b Obstetrics and Gynecology Hospital, State Key Laboratory of Genetic Engineering, Institute of Reproduction and Development, Children's Hospital, Fudan University, Shanghai 200011, China

Alzheimer's disease (AD) remains clinically incurable, raising a significant public health issue, particularly with its continuously increased incidence.^1^ Inflammatory bowel disease, including ulcerative colitis and Crohn's disease (CD), is characterized by long-standing inflammation and immunological imbalance of the gastrointestinal system.^2^ Recent epidemiological studies suggest a link between increased AD risk and inflammatory bowel disease.^3^^,^^4^ Due to the unclear regulatory mechanism potentially connecting AD with inflammatory bowel disease, this study is designed to reveal the underlying mechanisms at the genetic level and identify diagnostic markers to predict the risk of developing AD in patients with CD. Gene expression data of AD (GSE109887) and CD (GSE95095) were obtained from the GEO database for further bioinformatics analyses, including differentially expressed gene (DEG) identification, weighted gene co-expression network analysis, protein–protein interaction network construction, enrichment analyses, and immune infiltration. Least absolute shrinkage and selection operator (LASSO) regression was employed to filter hub genes in conjunction with another machine learning algorithm, random forest. Then, a nomogram was constructed and validated to assess the diagnostic value. Consequently, we identified four hub genes (*PDGFRB*, *BCL6*, *DDIT4L*, *TMEM106A*) strongly related to AD with CD, and constructed a credible diagnostic model based on these genes. Distinct but interrelated pathways were revealed in the pathological process of AD with CD, providing new perspectives on the molecular mechanisms linking these two diseases and underscoring the importance of further investigation of these shared genetic pathways. Moreover, the developed nomogram could assist clinical decision-making and highlight potential therapeutic targets for AD in CD patients. Figure S1 provides a concise overview of our research process.

We first identified 43 common DEGs (co-DEGs) with the same expression patterns (36 up-regulated and 7 down-regulated DEGs) based on GSE109887 and GSE95095 using the criteria of “fold change >1.5 and *P* < 0.01” (Fig. S2A–D; Fig. 1A). We then constructed a protein–protein interaction network to clarify the intricate interactions between proteins corresponding to the co-DEGs. This network was composed of 20 nodes and 19 edges, highlighting the pivotal roles of SPP1, PDGFRB, and BGN as hubs with a higher degree of connectivity compared with other nodes within the network (Fig. 1B; Fig. S3A). KEGG and GO analyses showed that co-DEGs were important for both AD and CD (Fig. 1C; Fig. S3B–D).

**Figure 1:**
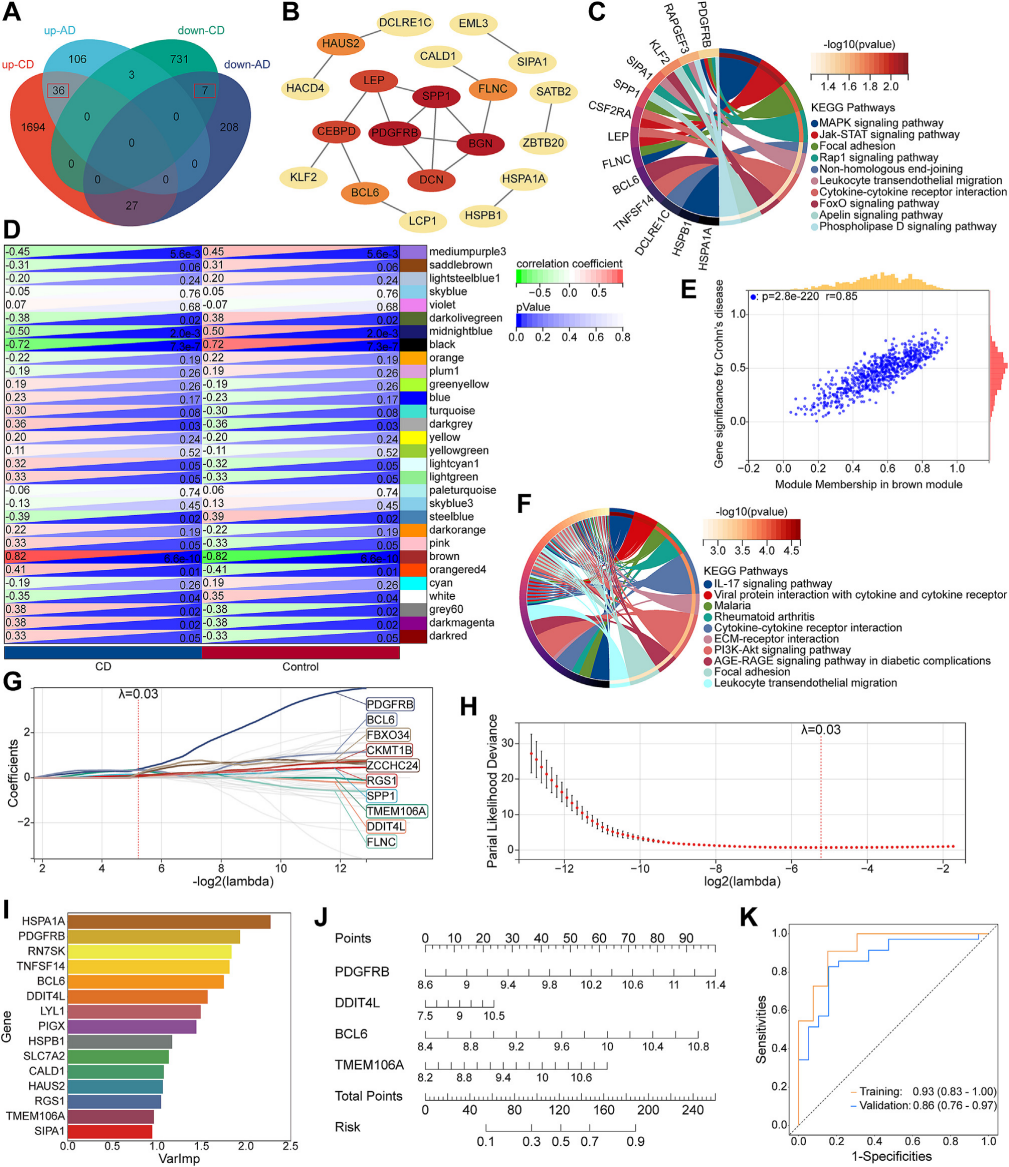
Identification of hub genes in diagnosing Alzheimer's disease with Crohn's disease by integrated bioinformatics analysis and machine learning. **(A)** Venn diagram of co-DEGs in AD and CD. The red boxes indicate DEGs with the same expression pattern. **(B)** The protein–protein interaction network of co-DEGs. **(C)** KEGG analysis of co-DEGs. **(D)** Correlation heatmap between modules and clinical characteristics. The upper left and lower right numbers in each cell represent the correlation coefficient and *p*-value, respectively. **(E)** Correlation of gene significance with brown module genes. **(F)** KEGG analysis of key genes in CD. **(G)** LASSO regression coefficient profiles. **(H)** Tuning parameter selection for LASSO. **(I)** Random forest analysis. Candidate genes are ranked by the importance score. **(J)** Nomogram construction for diagnosing AD with CD. **(K)** Receiver operating characteristic (ROC) curves of training set and validation set. AD, Alzheimer's disease; CD, Crohn's disease; DEG, differentially expressed gene; co-DEG, common DEGs.

Next, a scale-free co-expression network was created to find the module most relevant to CD (Fig. S4A–E). The correlations between CD and modules were computed (Fig. 1D). As a result, the brown module demonstrated the strongest positive correlation with CD (*P* = 6.6e-10, *r* = 0.82), considered a key module for the following enrichment analysis. Moreover, correlation analysis revealed a significant positive correlation (*P* = 2.8e-220, *r* = 0.85) between genes in the brown module and gene significance for CD (Fig. 1E). A total of 509 genes highly correlated with the pathogenesis of CD were obtained by taking the intersection of brown module genes from weighted gene co-expression network analysis (790 genes) and DEGs of CD dataset (2498 genes) (Fig. S5A). Based on the intersection of genes, KEGG and GO analyses enriched pathways that were highly associated with inflammatory response and immune response (Fig. 1F; Fig. S5B–D).

To construct a diagnostic model for AD with CD, we performed random forest and LASSO regression to screen for hub genes at first. The LASSO regression computed 10 potential diagnostic genes with *λ* = 0.03 (Fig. 1G, H), whereas the random forest evaluated and ranked candidate hub genes after calculating the variation importance (Fig. 1I). The 15 highest-ranking genes identified by random forest were intersected with the 10 candidate diagnostic genes from LASSO, and five genes (*PDGFRB*, *DDIT4L*, *BCL6*, *TMEM106A*, *RGS1*) were identified. *RGS1* was removed for further nomogram construction as it did not pass the single-factor logistic regression. We then calculated the area under the curve (AUC) and 95% confidence interval (CI) of the remaining four hub genes while plotting receiver operating characteristic (ROC) curves (Fig. S6A–D). The following are the detailed results: *PDGFRB* (AUC: 0.86, CI: 0.77–0.94), *DDIT4L* (AUC: 0.84, CI: 0.76–0.93), *BCL6* (AUC: 0.85, CI: 0.76–0.94), *TMEM106A* (AUC: 0.81, CI: 0.71–0.91). These findings demonstrated that the four candidate genes were highly valuable for diagnosing AD with CD. Therefore, we created a nomogram using the remaining key diagnostic genes (Fig. 1J) and then assessed the diagnostic efficacy of the newly established nomogram by constructing ROC curves (Fig. 1K). The following are the calculated AUC and 95% CI of each dataset: training set (AUC: 0.93, CI: 0.83–1.00), validation set (AUC: 0.86, CI: 0.76–0.97). The calibration plots of the training (*P* = 0.573) and validation (*P* = 0.772) dataset indicated a highly reliable predictive power of the nomogram (Fig. S6E, F). Collectively, all four candidate genes and the nomogram exhibited significant diagnostic value for AD with CD.

For a clearer understanding of immune regulation in AD with CD, we executed an immune infiltration analysis. The percentage of multiple immune cells differed significantly between AD and control samples, as well as between CD and control samples (Fig. S7A–D). Meanwhile, the up-regulated expression of hub genes was significantly associated with the infiltration level of multiple immune cells. Most notably, plasma cells were found to have a negative correlation with the expression of each hub gene (Fig. S7E, F). Moreover, gut-derived plasma cells are related to the inhibition of neuroinflammation,^5^ indicating a potential target for the immunotherapy of AD in patients with CD.

In summary, we obtained four hub genes through comprehensive bioinformatics methods and machine learning algorithms. *PDGFRβ*, *BCL6*, *DDIT4L*, and *TMEM106A* each play distinct yet interrelated roles in maintaining central nervous system homeostasis, regulating immune responses, and mediating inflammatory processes, all of which are crucial for elucidating the pathophysiology of AD and CD. The precise function of hub genes underlying AD development has been scarcely studied. Specifically, the reduction of PDGFRβ is linked to higher deposition of Aβ protein and reduced oxygenation. The PDGFRβ cells efficiently sense and transmit inflammatory signals from the peripheral system to the central nervous system, mediating neuroinflammation in multiple brain regions. BCL6 is essential for proper cortical neurogenesis by inhibiting Notch signaling in a Sirt1-dependent manner, which is a strong protective factor of AD. The dysfunction of DDIT4L was found to mediate the onset of AD-like symptoms through incorrect interactions with known pathogenic molecules of AD. Besides, TMEM106A may participate in the progression of AD by regulating the function of macrophages. Based on these four candidate diagnostic genes, we developed a diagnostic nomogram to support clinical deliberation. Furthermore, understanding their interactions and precise regulatory mechanisms offers potential therapeutic targets for treating AD in CD patients.

## Funding

This work was supported by the Key R&D Program of the Science and Technology Ministry of China (No. 2021YFC2701100) and the 10.13039/501100001809National Natural Science Foundation of China (No. 82150008, 81930036).

## Author contributions

**Pei Liu:** Writing – review & editing, Investigation, Formal analysis, Conceptualization. **Xiaoying Yao:** Writing – review & editing, Conceptualization. **Hongyan Wang:** Writing – review & editing, Project administration, Funding acquisition, Conceptualization.

## Conflict of interests

The authors declared no conflict of interests.
