# Supplementary material for: Hub genes identification for diagnosing Alzheimer's disease in patients with Crohn's disease
Source: Genes Dis. 2024 Nov 30;12(3):101476. doi: 10.1016/j.gendis.2024.101476 (PMC11803208; doi:10.1016/j.gendis.2024.101476)
Supplement: Multimedia component 1 [file mmc1.docx]

**Supplementary data**

**Materials and methods**

**Data acquisition and processing**

AD dataset (GSE109887) and CD dataset (GSE95095) were sourced from Gene Expression Omnibus (GEO) database**.** GSE109887, annotated by GPL10904, was generated from brain tissue samples of 46 AD patients and 32 ND controls. GSE95095, annotated by GPL14951, included 24 CD samples and 12 ND samples. Samples of GSE95095 dataset were obtained from intestinal tissue. The “limma” R package was applied to analyze differentially expressed genes (DEGs) of both GSE109887 and GSE95095, with criteria set at “fold change (FC) > 1.5 and *p* < 0.01”.

**Functional enrichment analysis and PPI network construction**

The “clusterProfiler” R package was applied to execute enrichment analysis with a criterion of *p* < 0.05. Kyoto Encyclopedia of Genes and Genomes (KEGG) and Gene Ontology (GO) analyses were each conducted twice based on different intersections of gene groups. STRING database was applied to establish the protein-protein interaction (PPI) network, with a 0.4 minimum interaction score. Visualization of the network was performed by Cytoscape software after removing nodes without node degree.

**Module gene selection of CD**

The “WGCNA” R package was applied to build a weighted gene co-expression network, enabling the exploration of correlation between expressed genes and their association with disease status. An optimal soft-thresholding power (β) was computed to construct this network according to the criterion of R^2^ > 0.85. Next, hierarchical clustering was utilized to construct modules containing genes with identical expression modes, ensuring each module contained at least 50 genes. Finally, we calculated the module eigengenes and identified their correlations with clinical features. The module exhibiting the highest coefficient of correlation with disease status was then chosen for further analysis.

**Machine learning**

Least absolute shrinkage and selection operator (LASSO) algorithm, implemented via “glmnet” R package, was applied to prevent overfitting and improve predictive accuracy. Random forest (RF) is another machine learning algorithm implemented via the “randomForest” R package. Finally, the overlapping genes of LASSO and RF were selected for further diagnostic model construction.

**Construction and validation of the nomogram**

We randomly divided the AD dataset into validation set and training set using a division ratio of 3:7. A nomogram was created with the “rms” R package to evaluate the diagnostic potential of these hub genes. The validation set was used for assessment of the newly established nomogram. Then, the receiver operating characteristic (ROC) curves of both datasets were plotted separately by the “pROC” R package. The calibration plots were constructed to visualize comparison between nomogram prediction and observed outcomes, which were assessed by the Hosmer-Lemeshow test with a criterion of *p* > 0.05.

**Immune cell infiltration analysis**

The “Cibersort” R package was used to conduct immune cell infiltration analysis. The proportion of various immune cell types was demonstrated via bar plots, while box plots compared the proportions among different groups. The correlation among candidate diagnostic biomarkers and infiltrating immune cells was assessed using Pearson correlation analysis, and the results were displayed using the “corrplot” R package.

**
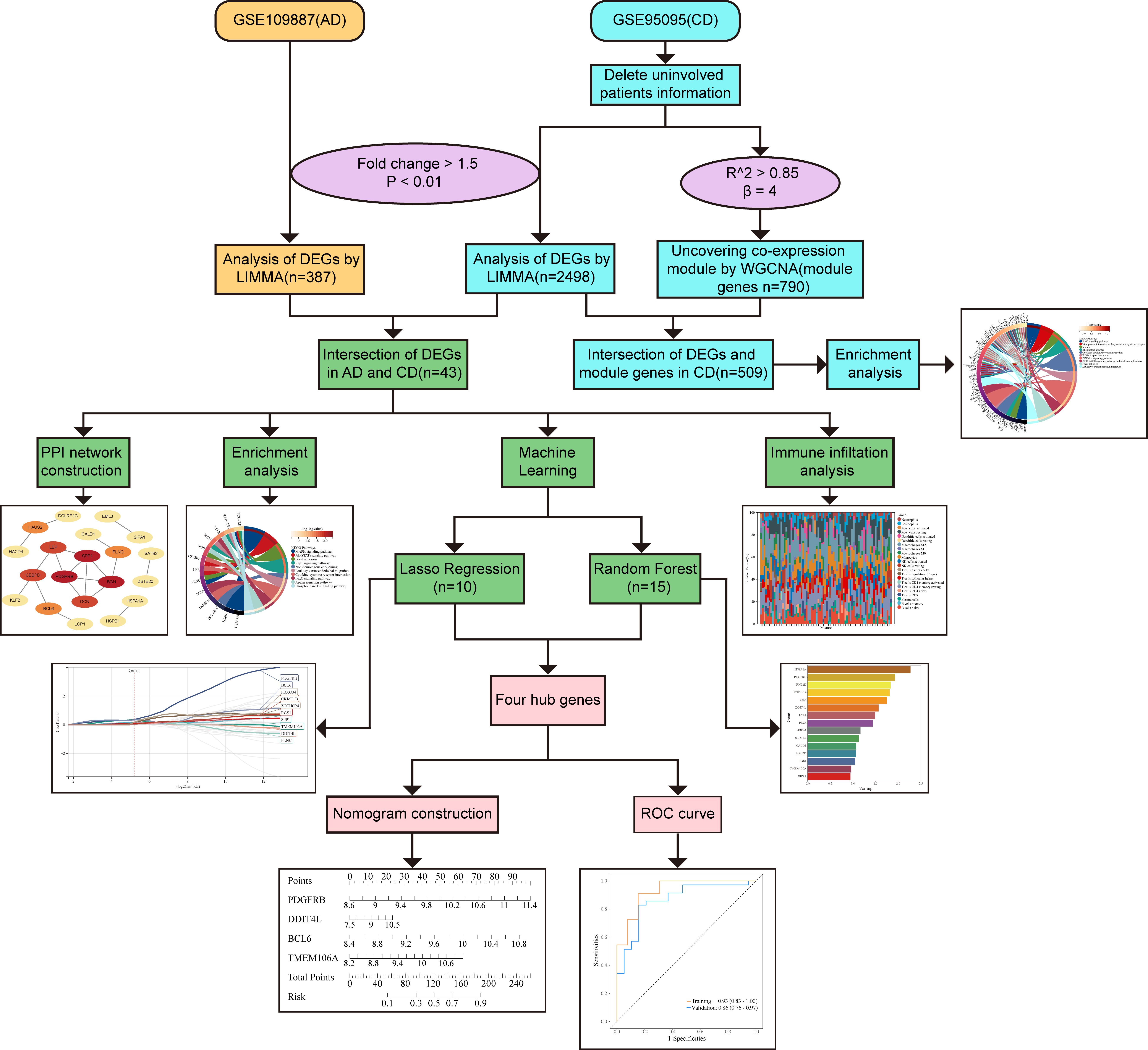
**

**Figure S1.** Flow chart of our research.


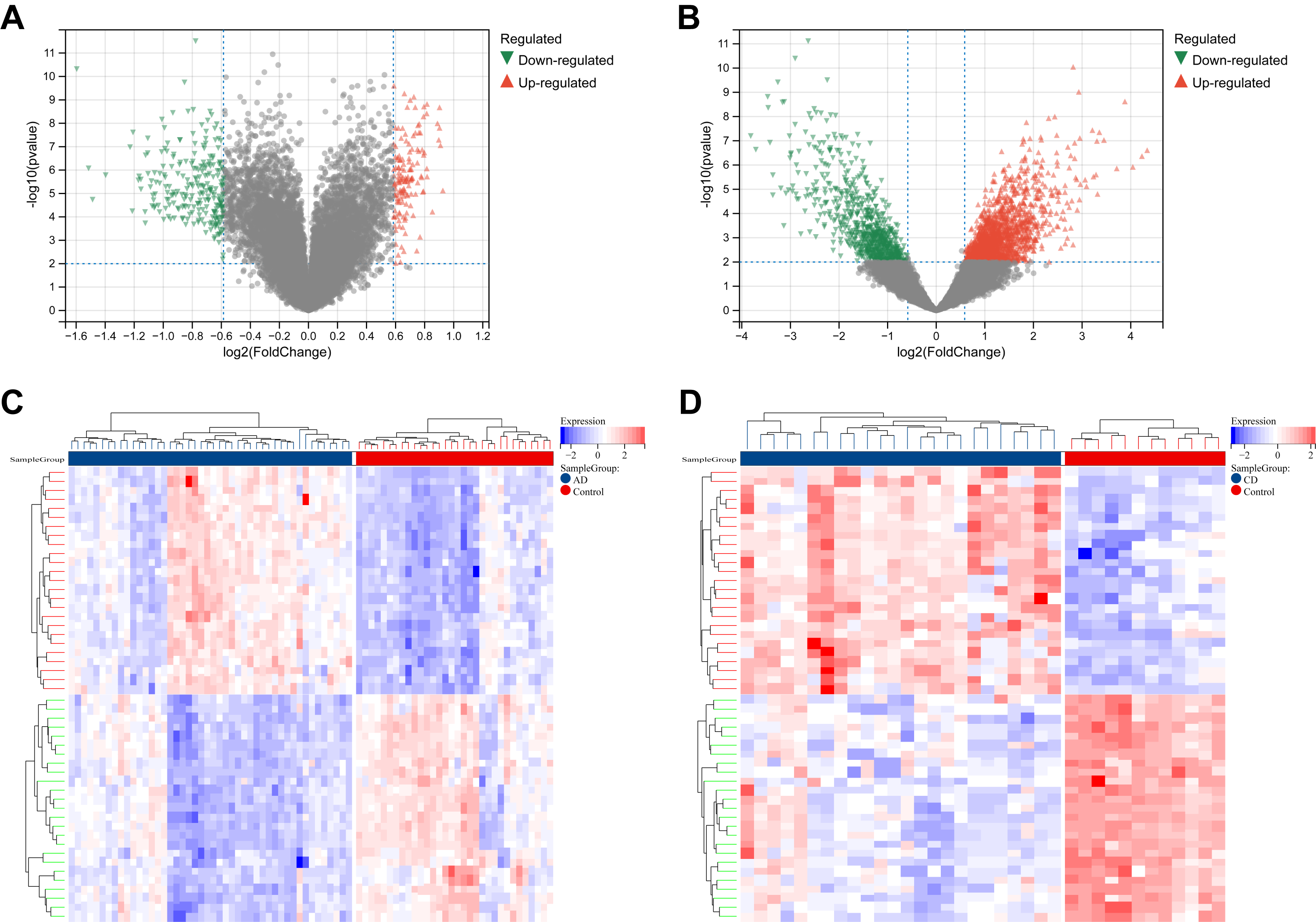


**Figure S2.** Screening of co-DEGs between AD and CD. **(A, B)** Volcano plots for AD, CD datasets. **(C, D)** Heatmaps for AD, CD datasets.


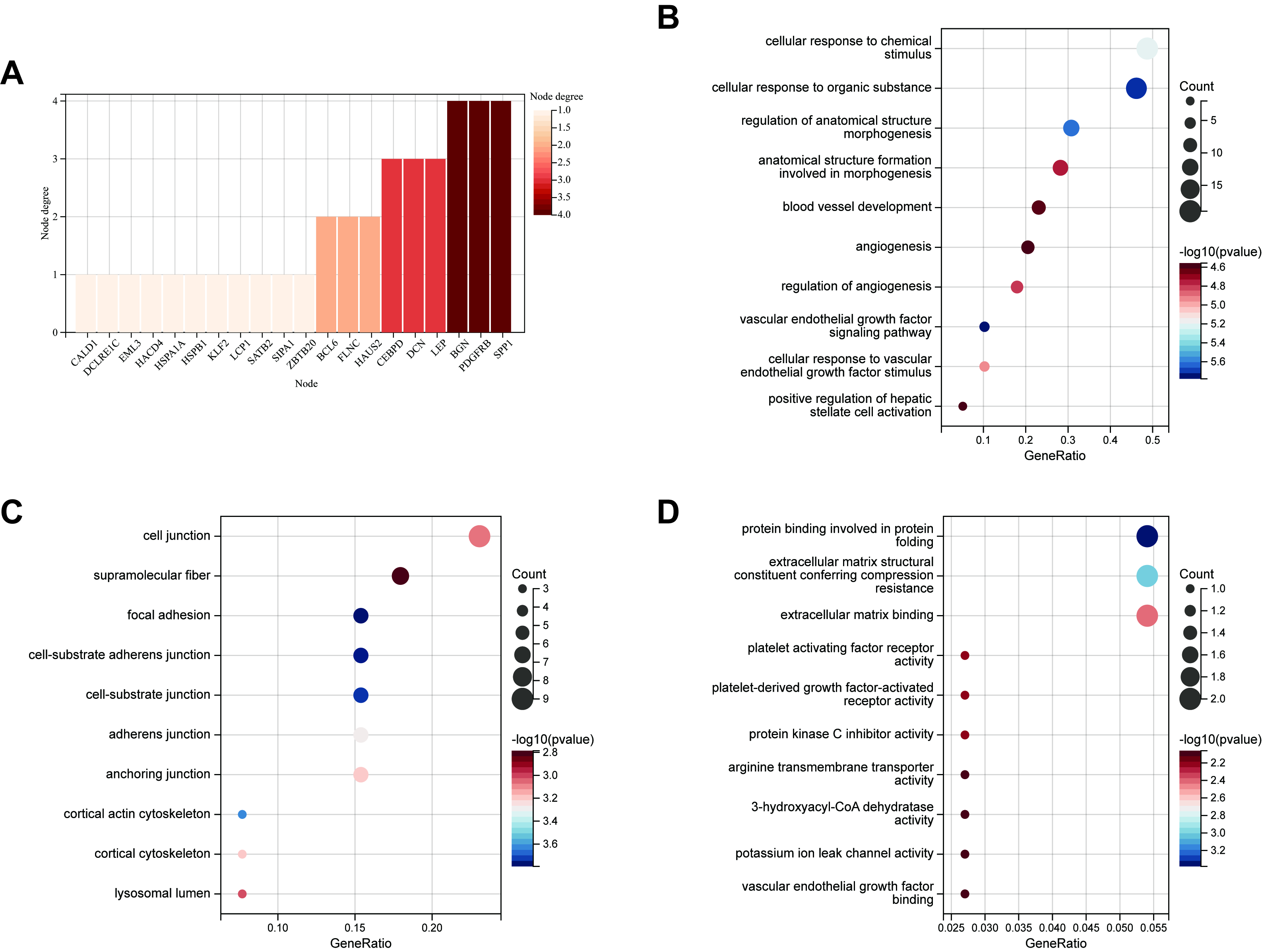


**Figure S3.** Enrichment analysis of co-DEGs and node genes recognition. **(A)** Histogram of node degree. **(B–D)** GO analysis (biological process, cellular component, and molecular function) of co-DEGs.


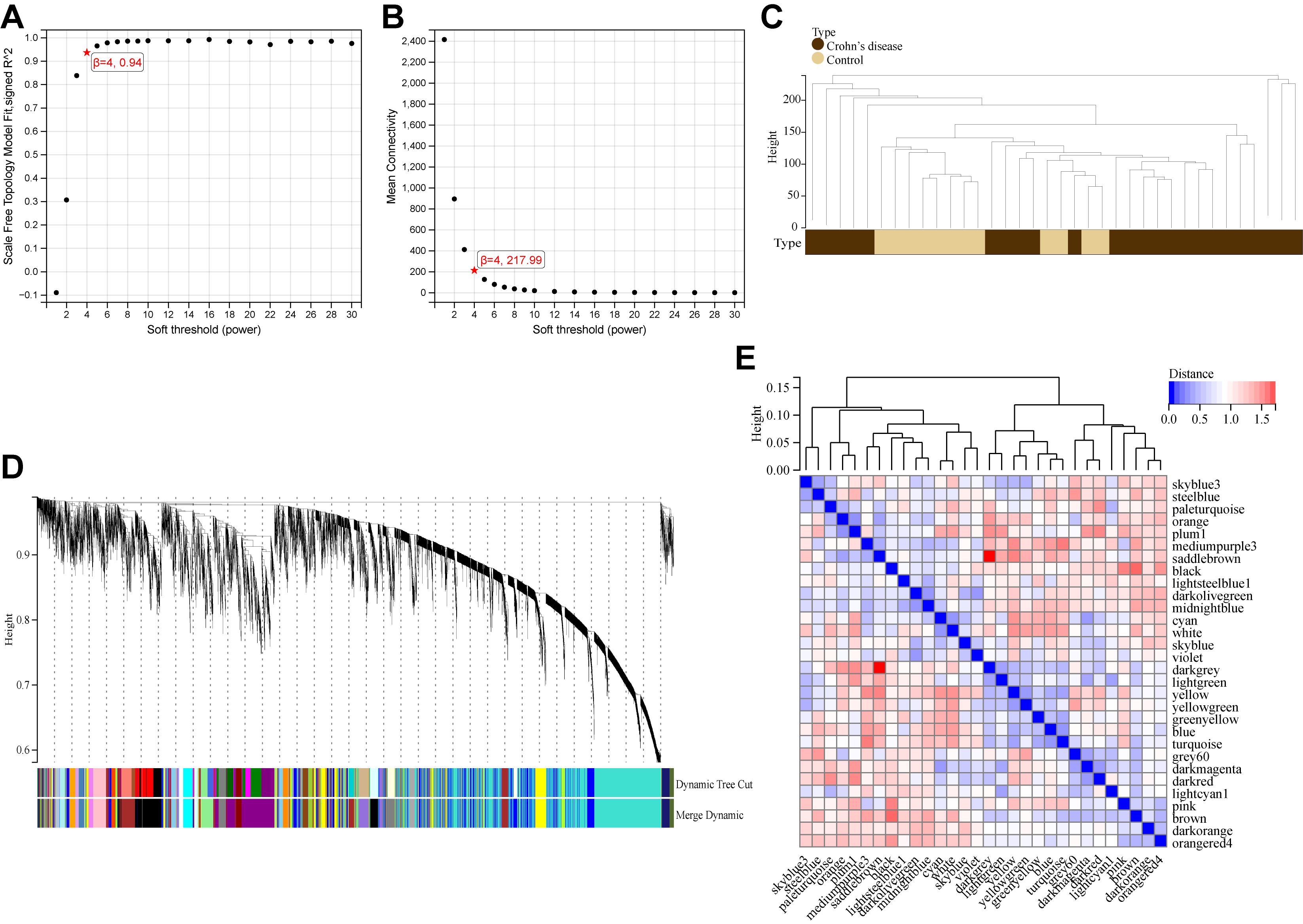


**Figure S4.** Identification of module genes in CD by WGCNA. **(A)** The scale independence. **(B)** The mean connectivity. **(C)** Dendrogram of CD and control samples. **(D)** Cluster dendrogram of co-expression genes. **(E)** Module eigengene adjacency heatmap.


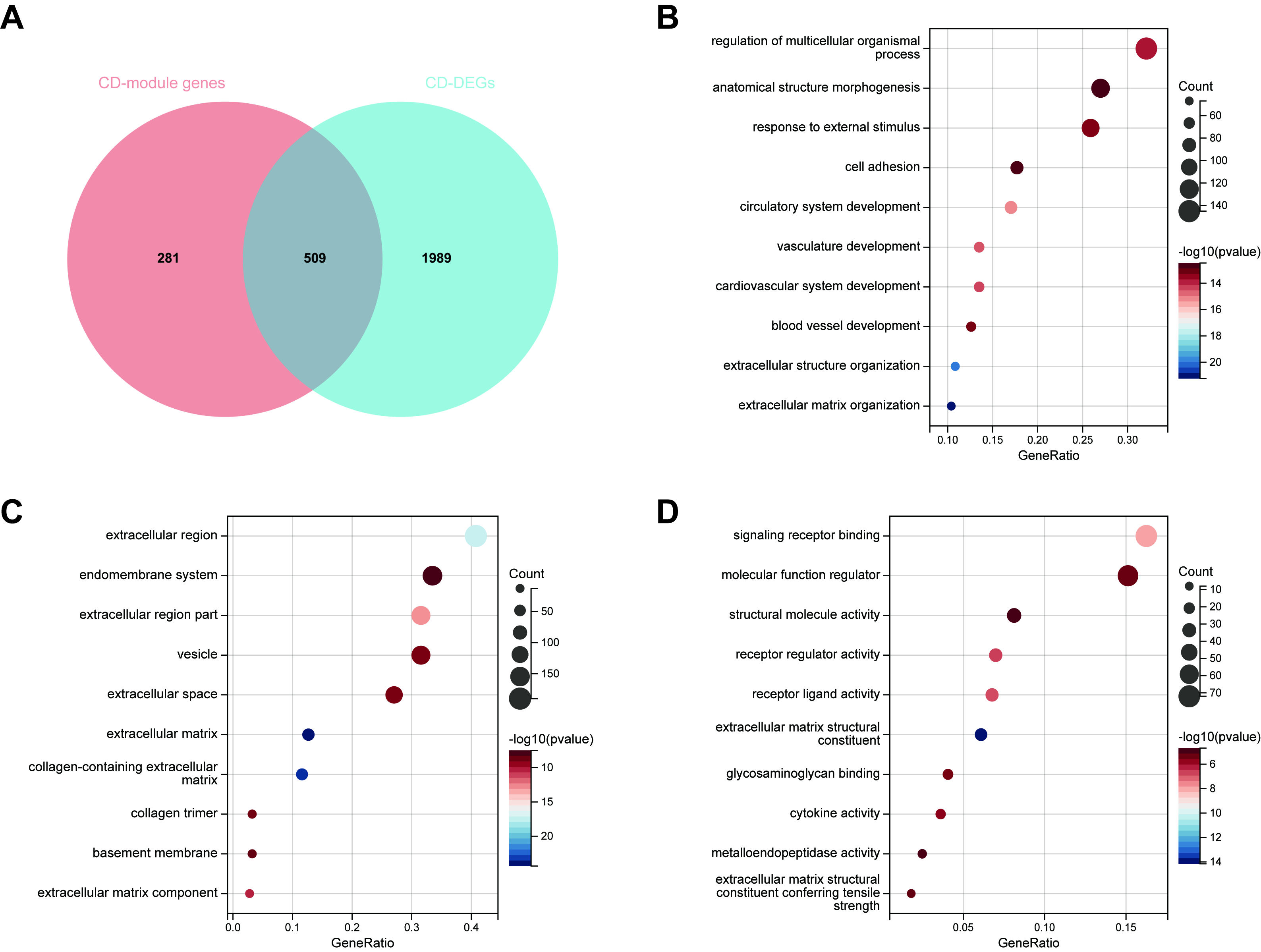


**Figure S5.** Enrichment analysis of key genes in CD. **(A)** Identify key genes via intersecting brown module genes and DEGs. **(B–D)** GO analysis (biological process, cellular component, and molecular function) of key genes in CD.


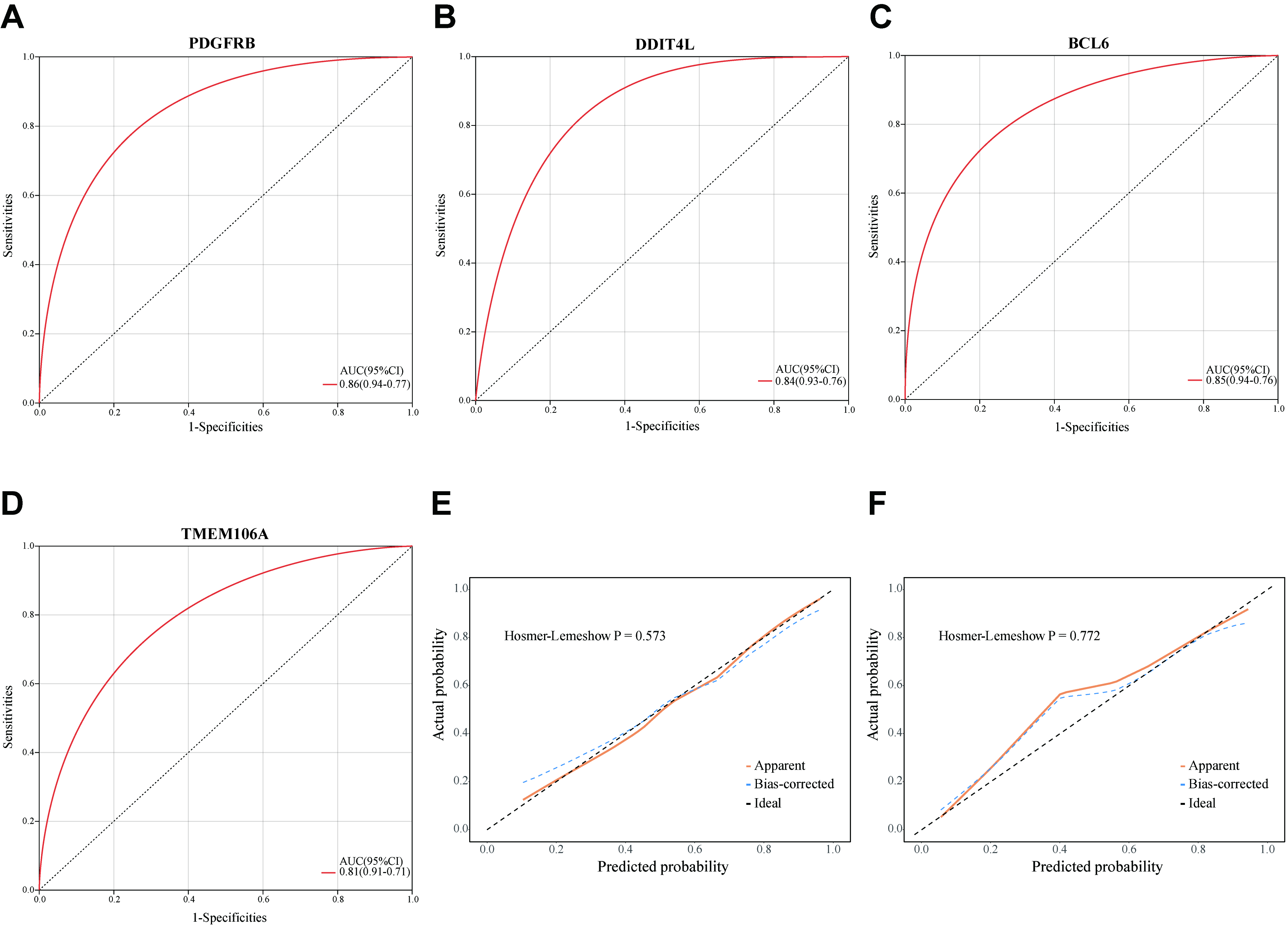


**Figure S6.** Hub genes identification for AD with CD via machine learning and evaluation of the diagnostic model. **(A–D)** The ROC curve of each hub gene (*PDGFRB*, *DDIT4L*, *BCL6*, and *TMEM106A*). **(E, F)** Calibration plots of training set and validation set.


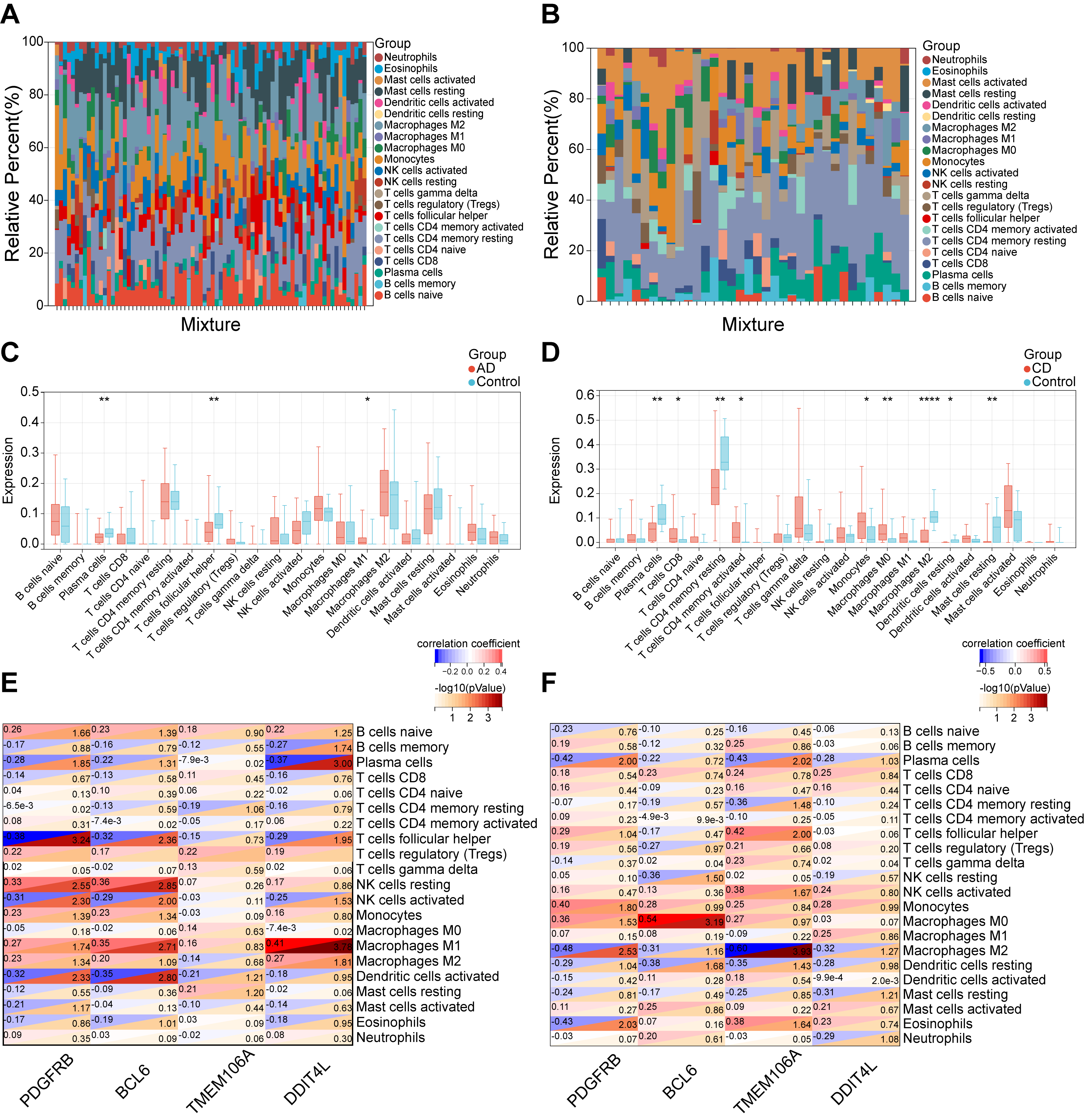


**Figure S7.** Immune infiltration analysis of AD and CD. **(A, B)** Column charts show the proportion of immune cells in each sample corresponding to GSE109887 and GSE95095. **(C, D)** Variations of immune infiltration in AD and control groups, CD and control groups. **P* < 0.05, ***P* < 0.01, *****P* < 0.0001. **(E, F)** Correlation heatmaps between hub genes and immune cells in AD and CD.
